# Effect of single-walled carbon nanotubes on tumor cells viability and formation of multicellular tumor spheroids

**DOI:** 10.1186/s11671-015-0858-7

**Published:** 2015-03-27

**Authors:** Olena M Yakymchuk, Olena M Perepelytsina, Alexey V Dobrydnev, Mychailo V Sydorenko

**Affiliations:** Department for Biotechnical Problems of Diagnostic, Institute for Problems of Cryobiology and Cryomedicine of NAS Ukraine, 42/1 Nauky str., 03028 Kiev, Ukraine; Department of Chemistry, Taras Shevchenko’ National University of Kiev, 60 Volodymyrska str., 01033 Kiev, Ukraine

**Keywords:** Carbon nanotubes, Multicellular tumor spheroids, Ultra dispersed diamonds

## Abstract

**Abstract:**

This paper describes the impact of different concentrations of single-walled carbon nanotubes (SWCNTs) on cell viability of breast adenocarcinoma, MCF-7 line, and formation of multicellular tumor spheroids (MTS). Chemical composition and purity of nanotubes is controlled by Fourier transform infrared spectroscopy. The strength and direction of the influence of SWCNTs on the tumor cell population was assessed by cell counting and measurement of the volume of multicellular tumor spheroids. Effect of SWCNTs on the formation of multicellular spheroids was compared with the results obtained by culturing tumor cells with ultra dispersed diamonds (UDDs). Our results demonstrated that SWCNTs at concentrations ranging from 12.5 to 50 μg/ml did not have cytotoxic influence on tumor cells; instead, they had weak cytostatic effect. The increasing of SWCNTs concentration to 100 to 200 μg/ml stimulated proliferation of tumor cells, especially in suspension fractions. The result of this influence was in formation of more MTS in cell culture with SWCNTs compared with UDDs and control samples. In result, the median volume of MTS after cultivation with SWCNTs at 100 to 200 μg/ml concentrations is 3 to 5 times greater than that in samples which were incubated with the UDDs and is 2.5 times greater than that in control cultures. So, if SWCNTs reduced cell adhesion to substrate and stimulated formation of tumor cell aggregates volume near 7 · 10^−3^ mm^3^, at the same time, UDDs reduced adhesion and cohesive ability of cells and stimulated generation of cell spheroids volume no more than 4 · 10^−3^ mm^3^. Our results could be useful for the control of cell growth in three-dimensional culture.

**PACS:**

61. 46 + w; 61.48 + c; 61.48De; 87.15-v; 87.64-t

## Background

**Figure 1:**
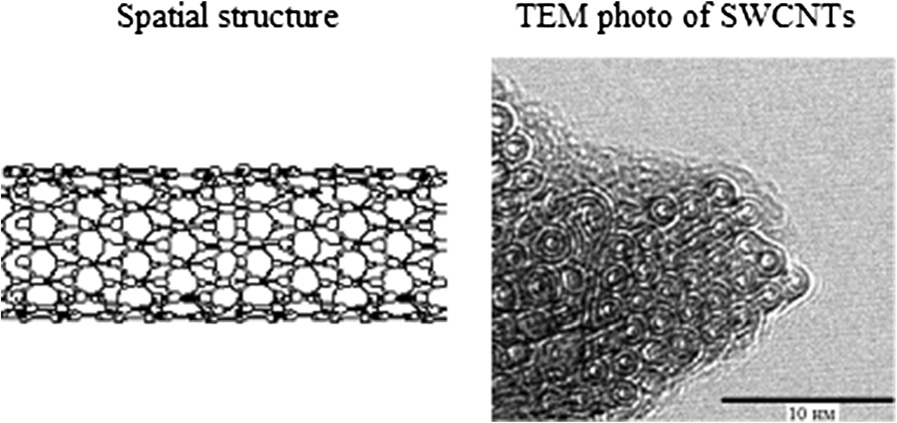
**Three-dimensional structure of the carbon nanotubes according to the literature** **[** 31 **,** 32 **].**

Single-walled carbon nanotubes (SWCNTs) are a unique structure. SWCNTs are usually produced by twisting two hexagonal fibers of graphite without seams. Nanotubes come in different forms: single-walled, multiwalled, straight, and spiral, with opened and closed ends. SWCNTs have a lot of unique properties. First, it has a high mechanical strength, which is a hundred times more strength than steel and provides the possibility to use it as probes in scanning tunneling microscopy. Nanotubes are extremely durable material like tensile and bending. Moreover, under the influence of mechanical stress exceeding the critical, nanotubes do not ‘rush’ and are tunable [1]. The nanotubes can be obtained simultaneously as a rugged and elastic tissue. The electrical properties of nanotubes are determined by their chirality [2,3]. Depending on chirality, single-walled tube can exhibit properties as graphite - semimetal [4,5]. Due to the small size of carbon nanotubes, the measurement of their electrical resistivity by four-pin way was conducted as early as in 1996. The resistivity of nanotubes can vary in considerable range. The minimal resistivity of nanotubes is much lower than that for graphite. Most of the nanotubes have metallic conductivity and properties of semiconductor with band gap from 0.1 to 0.3 eV (Figure 1). The researchers also opened another property of SWCNTs such as superconductivity [6]. Unique physical and chemical properties of nanotubes are complemented by extremely interesting biological properties. Carbon nanotubes (CNTs) have great potential for application in biology and medicine for bioimaging, targeted delivery vehicles for drugs, plasmid DNA, or small interfering RNA into cells by endocytosis and biosensors, and for mimicking extracellular matrix in tissue regeneration biotechnology. There are many efforts to employ CNTs as tool for anticancer therapy. In this field, carbon nanotubes were subject for fundamental works in cell biology. But there are a lot of unclear aspects of the interaction of nanotubes with tumor cells. Toxicity and carcinogenicity has been one of the major concerns for CNTs’ use in biomedical application. Therefore, in our study, we were looking for answers on a few questions. How do carbon nanotubes affect the survival of tumor cells in monolayer culture? Which properties of nanomaterial determine its relationship with the cells? Finally, which properties of nanotubes can be used in tissue biotechnology? To answer these questions, we chose a model of multicellular tumor spheroids.

Multicellular tumor spheroids or 3-D culture is a cellular system *in vitro*, which is regarded as a model for avascular tumor growth phase of tumor micronode formation [7,8]. Relevant literature suggests that this model combines the properties of many solid tumors such as: growth kinetics, cell heterogeneity, induction of proliferative signals, cell-cell interactions, development of specific histological structures, the level of secretion of signaling molecules, and the expression of antigens. Multicellular tumor spheroids are used in studies of basic biological mechanisms: regulation of proliferation, differentiation, apoptosis, invasion, angiogenesis, and cellular immune responses. At present, the main advantages of multicellular tumor spheroids (MTS) are their structure is well understood, the approach to the conditions is *in vivo*, and the morphological structure and biochemical parameters of neoplastic transformation of cells in the body are adequate. It has been shown that 3-D culture most correctly reflects the effect of cellular microenvironment on the development of tumor micronode *in vitro* [9,10].

So, the aim of the presented work is in investigation of cytotoxicity and biocompatibility of single-walled carbon nanotubes with tumor cell culture. Therefore, we studied how SWCNTs influence the formation of tumor micrometastases on the model of multicellular tumor spheroids.

## Methods

Single-walled carbon nanotubes for this study were kindly provided by the staff of G. V. Kurdyumov’s Institute for Metal Physics of the NAS of Ukraine. The material was purified by microwave-induced oxidation of carbon nanoparticles and subsequent washing with a 37 wt.% HCl. All solutions were prepared using Millipore water (Millipore, Billerica, MA, USA). In all experiments, 0.9% NaCl aqueous solution was used.

Ultra dispersed diamonds were produced by detonation of carbon-containing explosive by the commercial enterprise ‘Sinta’, Kharkiv reg., Ukraine. Individual ultra dispersed diamond (UDD) particles have sizes of 4 to 6 nm; the sizes of primary aggregates are approximately 20 to 30 nm with the specific surface area of approximately 550 m^2^/g [11].

### Cell line

Cell line of human Caucasian breast adenocarcinoma (MCF-7) was used for getting experimental model of tumor cell microaggregates. The line was purchased from Bank of Cell Lines and Tissues of Animals, Kavetsky’s Institute of Experimental Pathology, Oncology and Radiobiology NAS Ukraine. The cells were handled in standard tissue culture conditions (95% humidity, 5% CO_2_ in air; 37°C) under laboratory containment level 2.

### Fourier transform infrared spectroscopy

The chemical composition of the obtained nanomaterials was investigated by Fourier transform infrared spectroscopy (FTIR) spectroscopy. IR spectra were determined by FTS 7000e Varian FTIR spectrometer (Varian Medical Systems, Palo Alto, CA, USA). Samples for analysis were prepared by grinding in a mill of a mixture of approximately 1 mg of CNT and 150 mg of spectrally pure KBr. Samples were prepared by using a press with a pressure force of 3.0 to 3.5°10^3^ kg/cm^2^. Then, the samples were dehydrated by heating at a temperature of 600°C for 60 min. Pre-shot spectra of KBr were preliminary obtained; after that, they were subtracted from the spectra of the samples. All spectra were analyzed in accordance with the catalog of Spectrometric Identification of Organic Compounds [12].

### Preparation of stable suspensions of SWCNTs

The colloidal suspension of SWCNTs was carried out in two stages. In the first stage, carbon nanomaterials have been subjected to ultrasonic treatment in phosphate saline buffer (PBS) using an ultrasonic disperser UZDN - 2 T. The processing modes were *I* = 10 mA and *R* = 22 kHz, duration - 30 min. In the second stage, the resulting hydrosol was dispersed by the centrifugation at room temperature. The process includes several centrifugation cycles. So, the hydrophilic dissolving SWCNTs fraction was selected in this way. Before adding to the suspension cultured cells, solutions of SWCNTs were sterilized by boiling for 30 min.

### Three-dimensional cell model system

MTS MCF-7 cells (3-D culture) as model system of tumor micrometastasis was cultured by well-established method which was described earlier [13]. Briefly, cell suspension was counted using trypan blue and planted an equal number of cells (5°10^4^ cells/ml). The 3-D cell culture was maintained in RPMI medium (Sigma-Aldrich, St. Louis, MO, USA) with 10% FBS (Sigma-Aldrich, St. Louis, MO, USA) in standard conditions (95% humidity, 5% CO2 in air, 37°C). Generation of MTS was performed by technology developed in our laboratory. The cultivation of tumor cells were maintained for 24 h in 24-well plates coated with 1% agar in culture medium with 0.24% of carboxy-methyl-cellulose. For investigation, the dependence of the size and number of MTS on the concentration and type of nanomaterials (NM), MTS was generated in the presence of various concentrations of SWCNTs. The concentrations of nanomaterials were determined according to the forecasted therapeutic concentrations of drugs which could be established on the basis of these nanomaterials [14,15]. SWCNTs solution in PBS was added in the culture to the ultimate concentration of 12.5, 25, 50, 100, 150, and 200 μg/ml. After that, MTS generation was performed. Further cultivation was conducted for 48 h at a constant rotation of plates. At the next stage, microphoto images of MTS were taken by ‘dark field’ method. Overall, 120 images were done. Then, the volume of all MTS, which were on the files, was calculated. We used the formula of Rolf Bjerkvig: *V* = 0.4∙*a*∙*b*^2^, where *a* and *b* - the geometric sizes of the spheroids [16].

### Cell viability assay

To assay the concentration of planted cells and the ratio of alive and dead cells (MCF-7) after all variants of culturing, we counted the number of cells using 0.4% solution of trypan blue at standard procedure. To investigate the proliferation activity of cells MCF-7 after co-incubation with SWCNTs, we analyzed it with 3-[4,5-dimetltiazol-2]-2,5-dipheniltetratetrazolium (MTT) by colorimetric assay. MCF-7 cells, 1 · 10^4^, were seeded in a 96-well plate and incubated with SWCNTs 24 h. After that, we added 20 μl MTT solution (5 mg/ml PBS, Sigma-Aldrich, St. Louis, MO, USA) to 100 μl of cells suspension. Then, the cells were incubated with MTT for 4 h in standard conditions. Mitochondrial dehydrogenases of viable cells cleave the tetrazolium ring, yielding purple MTT formazan crystals which are insoluble in aqueous solutions. The resulting purple solution is measured by spectrophotometry. An increasing of cell number results in accumulation of MTT formazan and increasing in absorbance. Then, the samples were centrifuged under 1,500 *g* for 5 min and supernatant was removed. In all wells, 10 μl DMSO (Sigma-Aldrich, St. Louis, MO, USA) for MTT crystals dilution and 20 μl of 25 mM glycine were added. Optical absorption was detected on multiwell spectroscopy reader Multyscan (Labsystem, Helsinki, Finland) (540 nm).

### Statistical analysis, Pearson’s coefficient

For statistical analysis, all cell aggregates were sorted into groups according to MTS size from 1°10^−4^ to 1°10^−2^ mm^3^ with step in 1°10^−3^ mm^3^ and the estimated number and median of MTS volume for each group. For microstatistic assay normally distributed random variables, we used the Student’s coefficient for small population. To determine the relationship between concentration of NM and response of experimental biological systems, Pearson’s correlation coefficient was used. It was calculated for the median size of the cell spheroid culture and the concentration of SWCNTs and UDDs by formula [17]. The correlation coefficient ranges from ‘−1’ (inverse relation) to ‘+1’ (direct relation). Thus, for independent parameters, it is ‘0’ and for closely related approaches to the module unit.

## Results and discussion

### Chemical properties of nanomaterials

The chemical structure and purity of nanostructured materials which were used in experiments were determined by FTIR. Results were compared with literature data of FTIR spectra (Table 1.)

**Table 1:** **Compliance of FTIR analysis-based absorption peaks to types of chemical bonds in carbon nanomaterials** [11]

**Functional group**	**Type of vibration**	**Characteristic absorptions (cm** ^**−1**^ **)**	**Intensity**
Alcohol O-H	(Stretch, H-bonded)	3, 200 to 3,600	Strong, broad
Alkane C-H	Stretch	2,850 to 3,000	Strong
Acid O-H	Stretch	2,500 to 3,300	Strong, very broad
Aldehyde = C-H	Stretch	2,820 to 2,850 and 2,720 to 2,750	Medium, two peaks
Amine N-H	Stretch	3,300 to 3,500	Medium (primary amines have two bands; secondary have one band, often very weak)
Acid C = O	Stretch	1,700 to 1,725	Strong
Alkene C = C	Stretch	1,620 to 1,680	Variable
Aromatic C = C	Stretch	1,400 to 1,600	Medium-weak, multiple bands
Alkyl Halide C-F	Stretch	1,000 to 1,400	Strong
Ether C-O	Stretch	1,000 to 1,300 (1,070 to 1,150)	Strong
Ester C-O	Stretch	1,000 to 1,300	Two bands or more
Alkene = C-H	Bending	675 to 1,000	Strong

**Figure 2:**
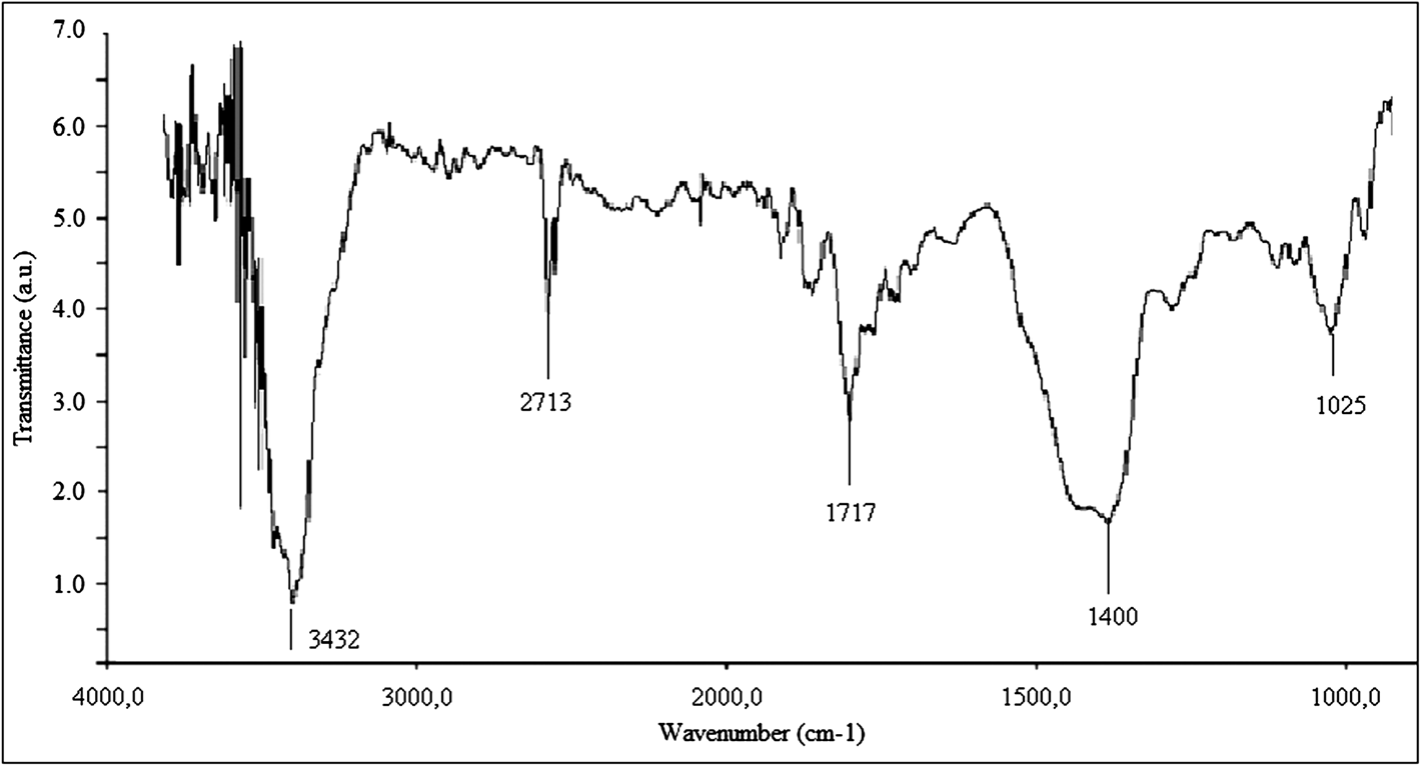
**FTIR spectra of SWCNTs.**

The results of SWCNTs FTIR assay are presented in Figure 2. The main features of SWCNTs (Table 1, Figure 2) correspond to the groups O-H stretched (3,600 to 3,200 cm^−1^). The amino N-H groups showed a strong signal at 3,431 and 3,411 cm^−1^. In SWCNTs samples, these were not vibration peaks of acid O-H (at 2,924 to 2,913 cm^−1^), alkane C-H (at 2,845 to 2,851 cm^−1^), and alkene = C-H groups (675 to 1,000 cm^−1^). Also, there was no absorption of the alkene C = C covalent band vibrations, which were fixed at 1,619 and 1,653 cm^−1^. Instead, there was quite intense peaks of acid C = O linkages in the region of 1,717 cm^−1^; it indicated the presence of carboxyl groups. Some C = O groups can emerge from adsorbed CO and CO_2_ (at 1,717 cm^−1^) in SWCNTs specters. The appearance of vibration at 1,400 cm^−1^ was the evidence of presence in SWCNTs samples of aromatic C = C carbon. Weak signals in the area of wave number 1,025 cm^−1^ were observed for CNT, which can be attributed to the presence of a small fraction of ether/ester C-O groups (Figure 2). In addition, according to the obtained spectra, SWCNTs have a small amount of absorbed or covalent chemical additives. Our spectra were similar to those presented by other authors [18,19], which allowed us to conclude that compliance with the chemical structure of the samples; their proper degree of purification are relevant for use in further biological studies.

### Biocompatibility of nanotubes in tumor cell culture

#### Biocompatibility of carbon nanotubes in tumor cell culture

**Figure 3:**
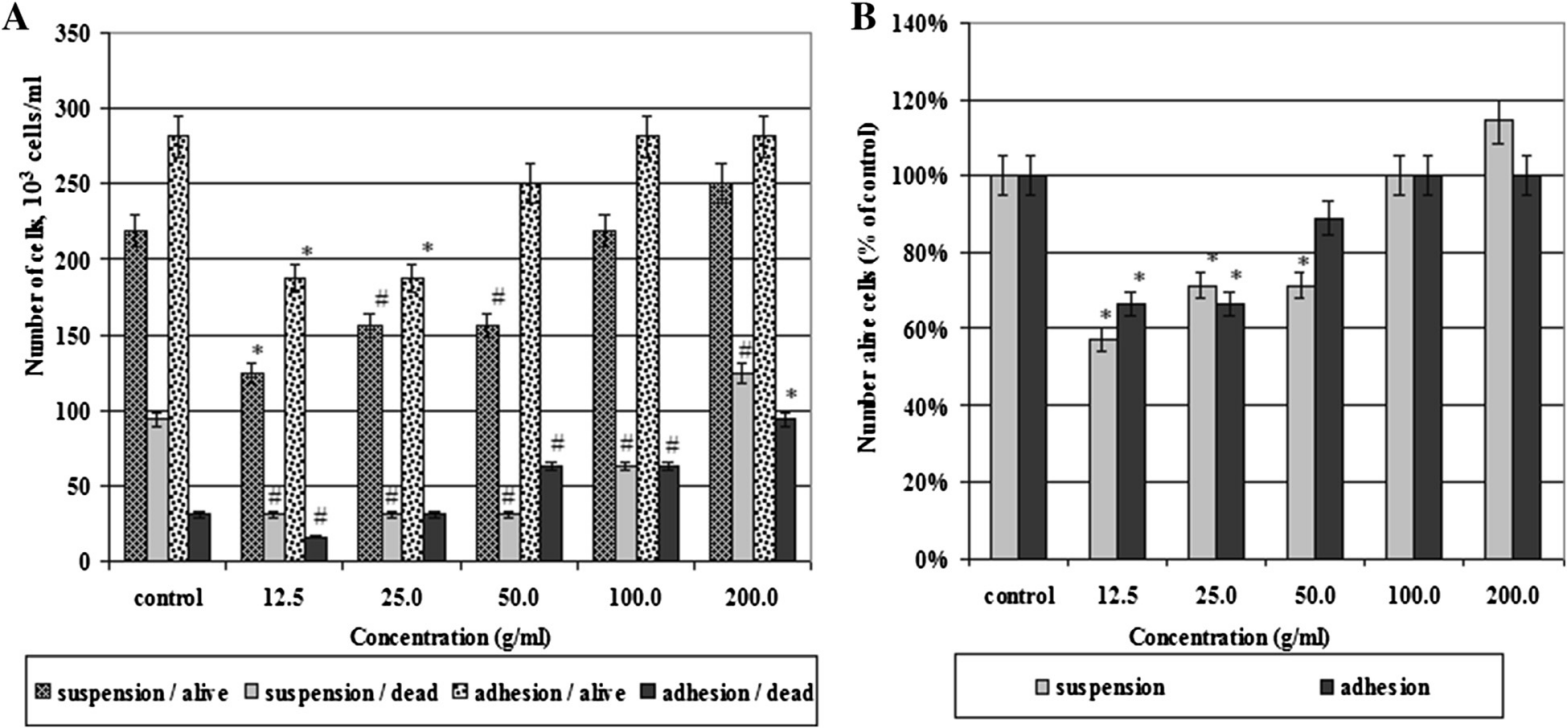
**Effect of SWCNTs on the number of alive and dead cells.** In adhesion and suspension fractions **(A)** and cell viability compare with control **(B)**. The number of alive/dead cells was counted due to usage of trypan blue. Cell viability was analyzed by MTT assay. *p < 0.01, #*p* < 0.05.

To determine the effect of SWCNTs on the cell survival and proliferation in culture, we incubated tumor cells MCF-7 at various concentrations of SWCNTs. After 7 days of incubation, we counted the number of alive and dead cells in adhesion and suspension fractions (Figure 3). As a result, it was found that small concentrations of SWCNTs (12.5, 25.0 μg/ml) had a cytostatic effect on tumor cells in adhesion as well as in suspension fractions (Figure 3A). We observed a reduction of the number of alive and dead cells in both fractions. It indicates a decreasing intensity of cell proliferation that led to a reduction of alive cell number at low concentrations of SWCNTs at 60% to 70% in comparison with the control (Figure 3B). As soon as tumor cells were cultured in the presence of higher concentration of SWCNTs (50 to 100 μg/ml), we watched the increase of alive cell number in adhesion and suspension fraction cell culture with simultaneous proportional increasing of number of dead cells (Figure 3A,B). The findings suggested that several processes occur simultaneously in the cell culture. On the one hand, increasing concentration of SWCNTs from 50 to 100 μg/ml and to 200 μg/ml leads to increasing proliferation of tumor cells both in adhesion and in suspension fractions. So if at 50 μg/ml of SWCNTs, the number of alive cells in adhesion is less than that in the control samples at 28.6%, at 100 μg/ml, the number of alive cells was equal to that in the control and at 200 μg/ml, it was more than that in the controls at 14.3%. The number of dead cells increased to a lesser extent. As a result, the percentage of alive cells along with increasing concentrations of SWCNTs in the adhesion fraction increased and reached a control level at 100 μg/ml with a further increase. On the other hand, the population of tumor cells in suspension at concentrations 50-100-200 μg/ml undergoes minimal cytotoxic effect. While the number of alive cells in suspension at 50 μg/ml was less than that in the control at 11.1%, at 100 and 200 μg/ml, it grows up to control parameters. This occurs due to both cell proliferation and cell migration from adhesion to suspension. We suggested that for suspension cell fraction, SWCNTs served as extracellular matrix and stimulated substrate independent cell viability. In subsequent experiments with high concentrations of SWCNTs, this assumption was confirmed (data not shown). Thus, the optimal concentrations of SWCNTs for cell incubation in our experiments were found equal to 100 μg/ml (for monolayer culture, 2-D) and 200 μg/ml (for multicellular tumor spheroids, 3-D).

### Effect of nanostructured materials for formation of multicellular spheroids

#### Effect of nanostructured materials on formation of multicellular tumor spheroids

In order to analyze the dependence of the size and number of MTS on the concentration and type of nanostructured materials, we generated tumor cell spheroids in the presence of SWCNTs. All cell aggregates were sorted into groups and estimated the number of MTS in each group as was described in the ‘Methods’ section.

**Figure 4:**
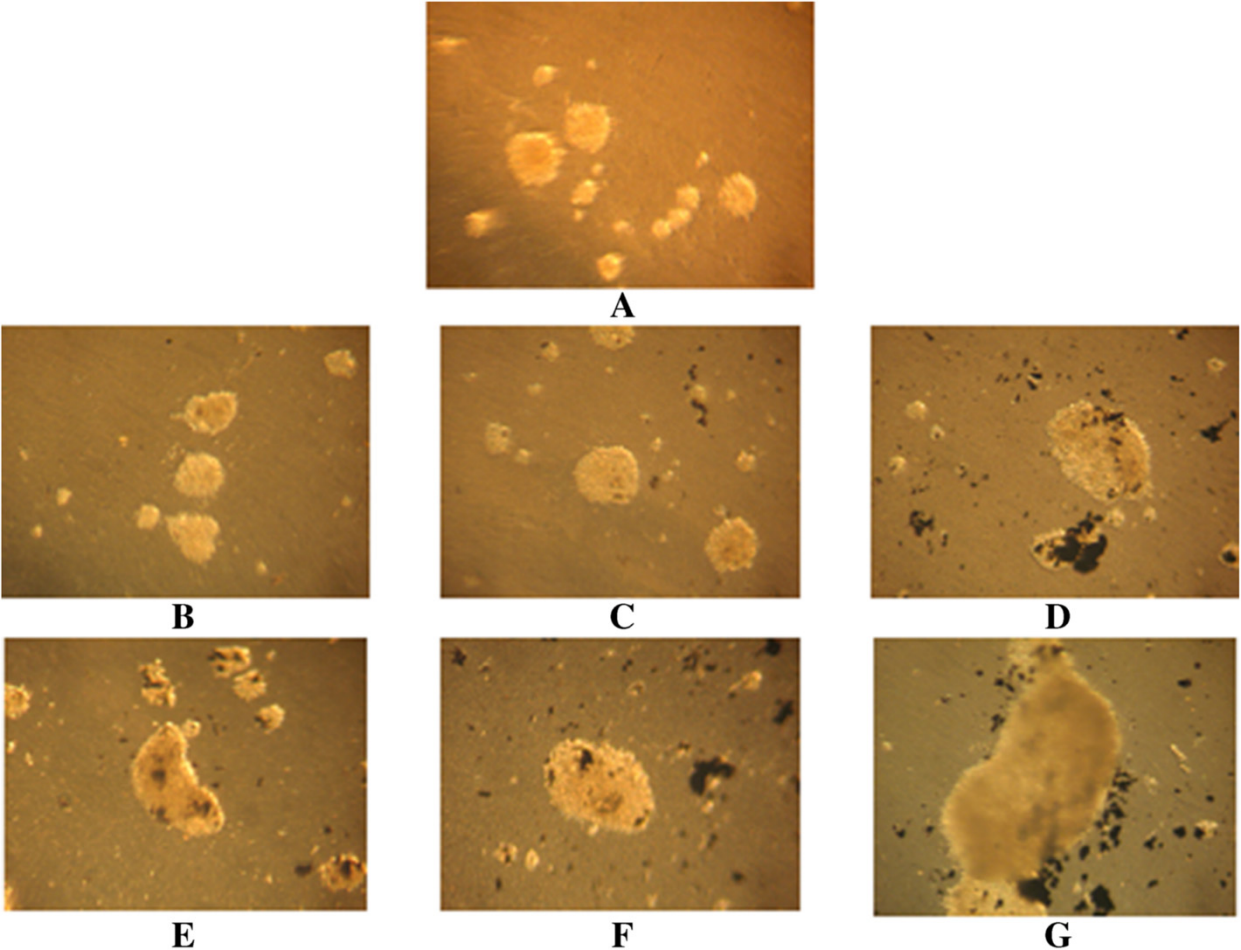
**Multicellular tumor spheroids culture at different concentrations of SWCNTs. (A)** Control. **(B)** 12.5 μg/ml. **(C)** 25 μg/ml. **(D)** 50 μg/ml. **(E)** 100 μg/ml. **(F)** 150 μg/ml. **(G)** 200 μg/ml. ×80 magnification.

**Figure 5:**
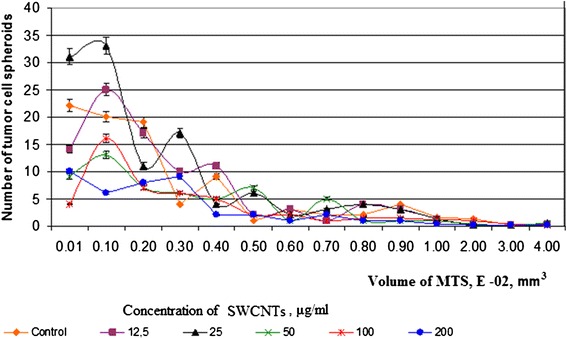
**Number of multicellular tumor spheroids at different concentrations of SWCNTs.**

**Figure 6:**
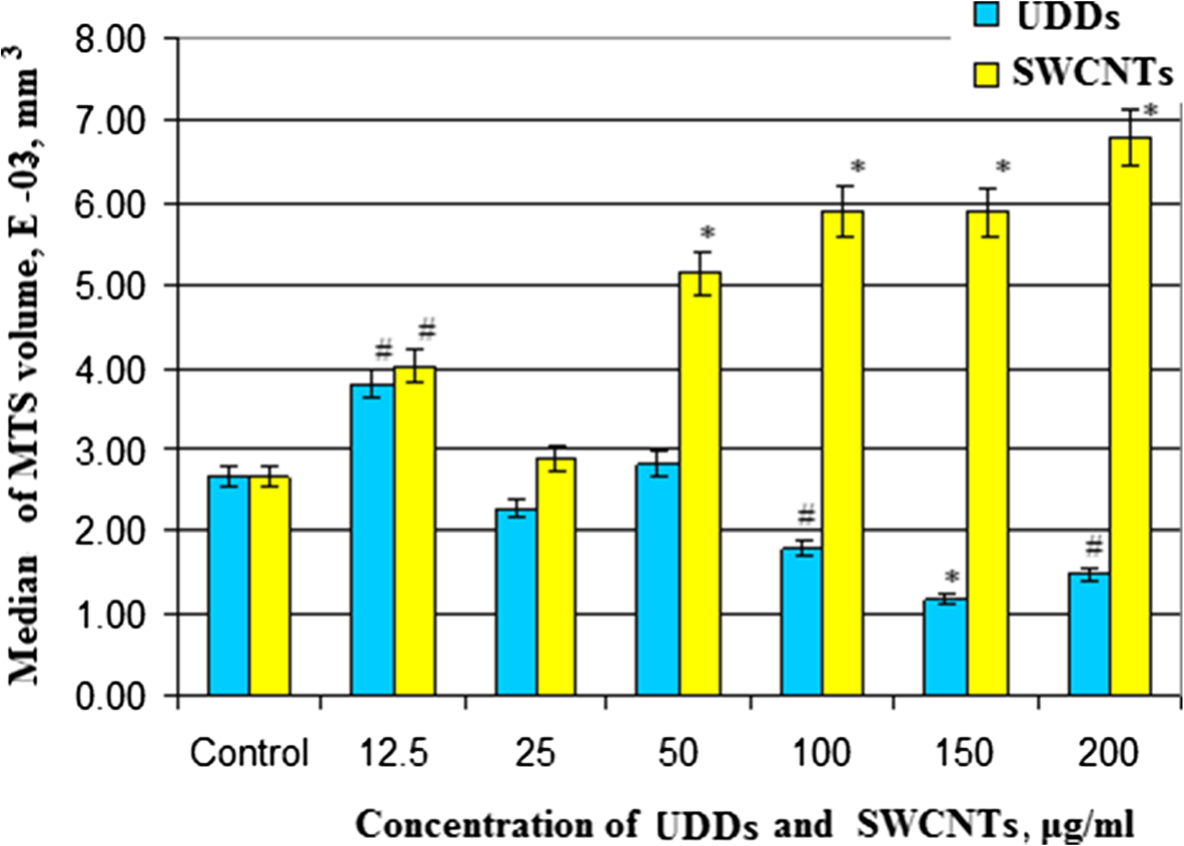
**Comparison influences of different concentrations of SWCNTs and UDDs on the formation of multicellular tumor spheroids.** *p < 0.01, #*p* < 0.05.

We discovered several physical, chemical, and biological trends. First, at low concentrations of SWCNTs (12.5 and 25.0 μg/ml), the highest number of small (10^−4^ mm^3^) tumor cell aggregates was formed (Figure 4). In the process of increasing concentrations of SWCNTs, we observed the growth of the volume of MTS and decrease of the number of it. So, small concentrations of SWCNTs stimulated cells to migrate into suspension and generate MTS with volume from 0.1 to 0.3°10^−4^ mm^3^. Increasing concentrations of SWCNTs from 50 to 200 μg/ml led to decrease the number of cell spheroids but enlarged their volume from 7 to 25°10^−2^ mm^3^. This trend was particularly notable at concentration of SWCNTs at 200 μg/ml. The viability of tumor cells in suspension fraction at 200 μg/ml of SWCNTs was even higher than that in the control. As a result, the volume of MTS was the largest at 200 μg/ml of SWCNTs. A positive correlation between concentration of SWCNTs and volume of MTS was confirmed by the statistical analysis. The Pearson’s coefficient in this series of experiments was 0.89. It means a very close positive correlation between concentration of SWCNTs and volume of MTS. So, we suggested that SWCNTs promoted cell migration, viability in suspension, and aggregation in spheroids. According to the literature, SWCNTs may act as an artificial extracellular matrix [20]. For example, Firkovska with colleges demonstrated that mouse fibroblast cells were able to grow on nanostructured carbon nanotubes substrates. In a recent series of experiments, they showed that the quality of the nanotubes can significantly stimulate the growth and proliferation of mammalian cells. These results showed that highly ordered arrays of carbon nanotubes can be used to manage and control the growth of mammalian cells [21]. Our data can be viewed from two perspectives. Firstly, incubation of tumor cells with certain concentrations of nanotubes clearly leads to tumor progression. This happens after stimulation of cell detachment from the substrate and the formation of large numbers of small tumor spheroids (micrometastases). As reported in the literature, cell aggregate of 0.1 mm in diameter already ables to migrate into the blood stream and lymphoid system and to form a secondary tumor [22]. In connection with this, oncogenic activity of the SWCNTs under certain conditions and prerequisites can cause cancer and should be the object of attention of scientists [23-25]. So nanostructured materials are perspective for creation of biological sensors, carriers of drugs, and diagnostic elements but should be used very carefully [26-28]. The effect of nanotubes on the development of tumor process can occur at the level of cell-to-cell interaction, with extracellular matrix and stoma structures. On the other hand, according to our research and literature data, chemical and physical properties of SWCNTs demonstrated low cytotoxic effect on the intracellular structures [11,29,30]. Therefore, the usage of SWCNTs as basis for intensive growth of cells in suspension, three-dimensional culture, and tissue-like structures seems very promising. Proof of this is in well-known biotechnology experiments which describe fibroblast-like and endothelial cells growth on thin films of SWCNTs [19]. Second, the trend influences the chemical properties of nanomaterials on the formation of MTS. To compare the effects of SWCNTs and UDDs on the formation of cell aggregates, we calculated the median of volume of MTS at appropriate concentrations of these nanostructures (Figure 5). The formation of MTS is very important because tumor spheroids are well-known model of avascular stage of tumor growth and formation of micrometastases. Another feature that caught our attention relates to the physical characteristics of NM, which affect the relationship between cells and carbon structure. For comparison, we used carbon structures called UDDs. We described their properties and effects on cells in another paper [11]. So, we compared the sizes of MTS which were formed in the presence of SWCNTs and UDDs. We found that UDDs stimulated the formation of MTS at concentrations of 12.5 to 50 μg/ml. Therefore, SWCNTs created more favorable conditions for cell spheroid growth at concentrations 100 to 200 μg/ml. At the same time, the median of MTS volume was larger in the presence of SWCNTs than that in the presence of UDDs at all concentrations (Figure 6). We calculated Pearson’s coefficient for UDDs. It was −0.81. So, UDDs demonstrated inverse correlation between the size of cell aggregates and concentration of UDDs. The explanation of this phenomenon is in the size of nanostructured aggregates, their effect on cell adhesion to the substrate, and physical properties of cells and NM. Nowadays, there are a lot of papers devoted to biological usage of SWCNTs and its promising prospects. But mechanisms of interaction of SWCNTs with living cells and tissues still remain unclear. It should be noted that use of SWCNTs in biological systems encounters on a number of difficulties. For example, the hydrophobic nature of SWCNTs as well as all carbon nanomaterials promotes fast aggregation of SWCNTs in physiological solutions. At the same time, the functionalization of SWCNTs provides better solubility and changes the electrochemical properties of substances. For example, according to the literature, zeta potential of SWCNTs is about −10 to −20 mV in pH range 6.0-8.0 [29]. It should be remembered that zeta potential of a healthy cell is about −20.5 mV. Processing SWCNTs in surfactant, for example, cetyltrimethylammonium bromide (CTAB) leads to change in zeta potential from −19.38 to 49.51 mV and better solubility [30]. According literature data three-dimensional and chemical structure of SWCNTs is quite complicated [31,32]. Our assay of SWCNTs FTIR spectra testified the presence of a small amount of O-H and N-H groups (lines in 3,432 nm area). Low power of functionalization and high removal of impurities provided aggregation of SWCNTs and formation of cohesive centers for tumor cells in three-dimensional culture. And the same negative zeta potential of cells and SWCNTs prevented the establishment of strong focal cellular contacts with cells and carbon aggregates. We observed a different trend for the UDDs. In our previous studies, we have demonstrated that the UDDs stimulated the formation of multicellular tumor spheroids at low concentrations. FTIR assay of UDDs demonstrated a presence of a large number of functional groups on its surface, and zeta potential was about +40 mV. This leads to a weakening of cell-substrate contact, as we observed at low concentrations of the UDD [11]. But this effect is not sufficient for the formation and survival of large cell aggregates in the suspension. We assumed that the differences in cell-to-cell interactions in culture and those with considered nanostructures can be explained by differences in electrochemical properties of nanomaterial substances and cells.

## Conclusions

As a result, we found that SWCNTs did not have cytotoxic effects on tumor cells in suspension and adhesion. At low concentrations, SWCNTs demonstrated cytostatic effect on MCF-7 cells. Increasing of SWCNTs concentration led to stimulation of proliferation of tumor cells and rising percentage of alive cells. This phenomenon was confirmed by the analysis of the number and size of MTS which were formed in the presence of SWCNTs. In MCF-7, cell culture generated a large number of MTS up to 1 · 10^−3^ mm^3^ volume at concentration of SWCNTs from 12.5 to 50 μg/ml. When the concentration of SWCNTs increased to 100 to 200 μg/ml, the number of MTS decreased. However, the size of the tumor aggregates increased to 7 · 10^−3^ mm^3^. We compared the effect of SWCNTs and UDDs on the formation of MTS. We demonstrated that SWCNTs created more favorable conditions for cell spheroid growth at concentrations 100 to 200 μg/ml, UDDs at 12.5 to 50 μg/ml. We suggested that zeta potential of the nanostructured substances has significant impact on their interaction with tumor cells. Zeta potential influences on the ability of carbon materials to form colloidal solutions, such as UDDs or to form agglomerates as SWCNTs. Moreover, it determines the establishment of mutual influence of nanomaterial-to-cell, cell-to-cell, and cell-to-substrate. So we suggested that carbon nanomaterials such as single-walled nanotubes are suitable base for artificial extracellular matrix. Particular low cytotoxicity and influence on intracellular structures of SWCNTs allow them to be useful for creation of three-dimensional cell culture. Unfortunately, SWCNTs may be a potential threat to tumor development due to its ability to stimulate cell migration and support cell viability without substrate. In this case, SWCNTs themselves perform the role of artificial extra cellular matrix. Nevertheless, our deep conviction is that if we know the nature of substance and its possible negative influence, we are able to avoid the detrimental effects of SWCNTs and use their positive biotechnological potential. Our finding could be applied quite reliably in other bioengineering context with maximum advantage for medicine.
